# Pediatric sigmoid colonic perforation with *Campylobacter* enterocolitis: a case report and review of the literature

**DOI:** 10.1186/s13256-022-03711-1

**Published:** 2022-12-30

**Authors:** Yung-Yu Chu, Cheng-Yi Lin, Tien-Lin Kuo, Shu-Chi Mu, Beng-Huat Lau, Yuh-Yu Chou

**Affiliations:** 1 School of Medicine, College of Medicine, Taipei Medical University, Taipei, Taiwan; 2 Department of Paediatrics, Shin Kong Wu Ho-Su Memorial Hospital, Taipei, Taiwan; 3 Medical College, Fu-Jen Catholic University, New Taipei, Taiwan; 4 Department of Pathology, Shin Kong Wu Ho-Su Memorial Hospital, Taipei, Taiwan

**Keywords:** *Campylobacter*, Pediatric, Sigmoid colonic perforation, Case report, Review of literature

## Abstract

**Background:**

*Campylobacter*-related infectious gastroenteritis is common and usually self-limited. Intestinal perforation is a rare complication of the infectious colitis caused by *Campylobacter*, and only handful of cases have been reported. This is the first published case report of pediatric *Campylobacter* intestinal perforation located in the sigmoid colon.

**Case presentation:**

A 15-year-old previously Taiwanese healthy boy presented with 5 days of fever up to 39.8 °C, with right lower quadrant abdominal pain and watery diarrhea. Although he received antimotility agents and antipyretics at a local clinic to relieve symptoms, he came to the emergency department with signs of shock manifesting as hypothermia to 35.2 °C, tachycardia, and low blood pressure. Laboratory testing demonstrated leukocytosis with left shift and significant elevation of C-reactive protein. Stool and blood cultures were obtained, and he was admitted for fluid challenge and antibiotic treatment. On the second day of admission, he suffered from sudden onset of severe, diffuse abdominal pain. Physical examination revealed muscle guarding, rebounding tenderness, and silent bowel sound. Abdominal X-ray showed subdiaphragmatic free air at standing view. The patient underwent emergent exploratory laparotomy, which revealed sigmoid colon perforation about 0.5 cm. Enterolysis and repair of sigmoid colon were performed. Intraoperative stool specimen nucleic acid amplification testing had turned positive for *Campylobacter* spp. with negative results for other bacterial pathogens. His symptoms improved and he tolerated food well, and was discharged 15 days after admission.

**Conclusions:**

We present this case because of the rarity of *Campylobacter*-induced sigmoid colon perforation in the pediatric population. It is important to keep in mind that sigmoid colon perforation can be due to an infectious cause, and one of the culprits can be *Campylobacter*. Infectious colitis caused by *Campylobacter* spp. should be managed cautiously and the use of antimotility agents in such conditions should be considered judiciously.

## Background

*Campylobacter* is a leading cause of infectious gastroenteritis that is usually self-limited. Although *Campylobacter* species infection can result in a variety of complications, rarely does it cause intestinal perforation. Sigmoid perforation is exceedingly rare. In this report, we present a case of enterocolitis secondary to *Campylobacter* spp. complicated by sigmoid perforation.

## Case presentation

**Fig. 1:**
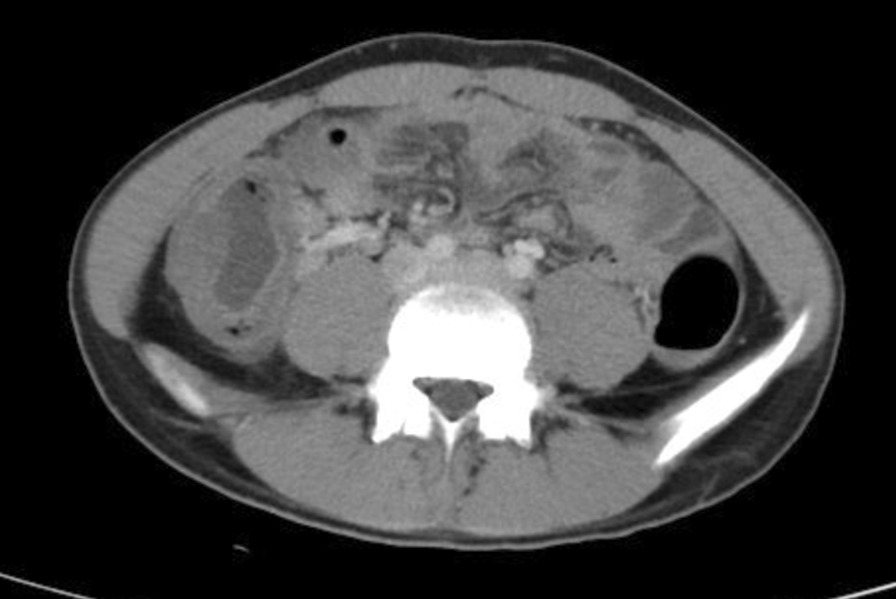
Non-contrast-enhanced computed tomography revealed acute enterocolitis consisting of moderately distended, thickened, fluid-filled loops of the ascending colon and transverse colon, and intramural air noted at the ascending colon

A 15-year-old previously Taiwanese healthy boy presented with 5 days of fever, right lower quadrant abdominal pain, and watery diarrhea after consuming abundant seafood. Stools were watery but no blood or mucus were noted. He was febrile up to 39.8 °C before the presentation. He went to the local clinic at first. Injection with buscopan (hyoscine-*N*-butylbromide) , loperamide, and antipyretics were prescribed to relieve symptoms. Although he stopped having diarrhea, his fever still lasted more than 2 days. He was presented to the emergency department with signs of shock [1]: he was hypothermic to 35.2 °C, tachycardic to 102 beats per minute, and with low blood pressure of 90/55 mmHg. A basic metabolic panel and complete blood count were remarkable for leukocytosis of 23,400/μL with left shift with 8% band form and significant elevation of C-reactive protein (CRP) of 39.57 mg/dL. Abdominal and pelvic computed tomography scans demonstrated acute enterocolitis consisting of moderately distended, thickened, fluid-filled loops of the ascending colon and transverse colon, and intramural air noted at the ascending colon (Fig. 1). Stool and blood cultures were obtained, and he was admitted for fluid challenge and antibiotic treatment.

**Fig. 2:**
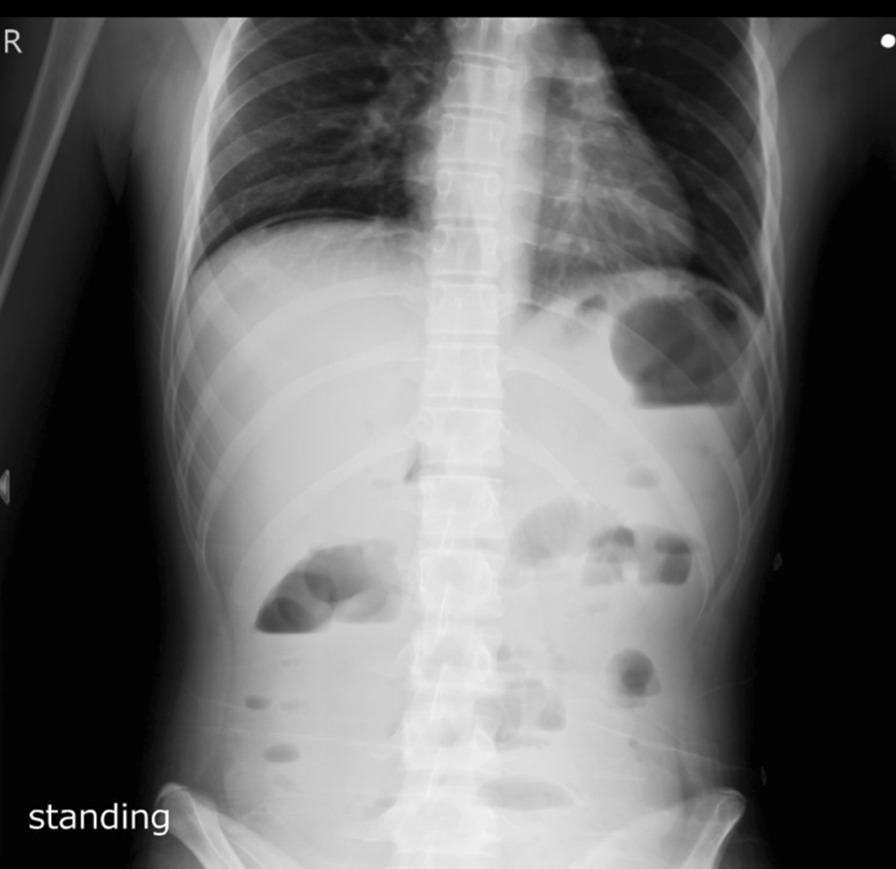
The standing view of abdominal plain film showed subdiaphragmatic free air

On the second day of admission, despite adherence to metronidazole, ceftriaxone, and vancomycin, his symptoms persisted along with low blood pressure with widened pulse pressure (100/45 mmHg). In the evening, he suffered from sudden onset of severe, diffuse abdominal pain. Physical examination revealed muscle guarding, rebound tenderness, and silent bowel sound. Abdominal X-ray showed subdiaphragmatic free air at standing view. (Fig. 2).

**Fig. 3:**
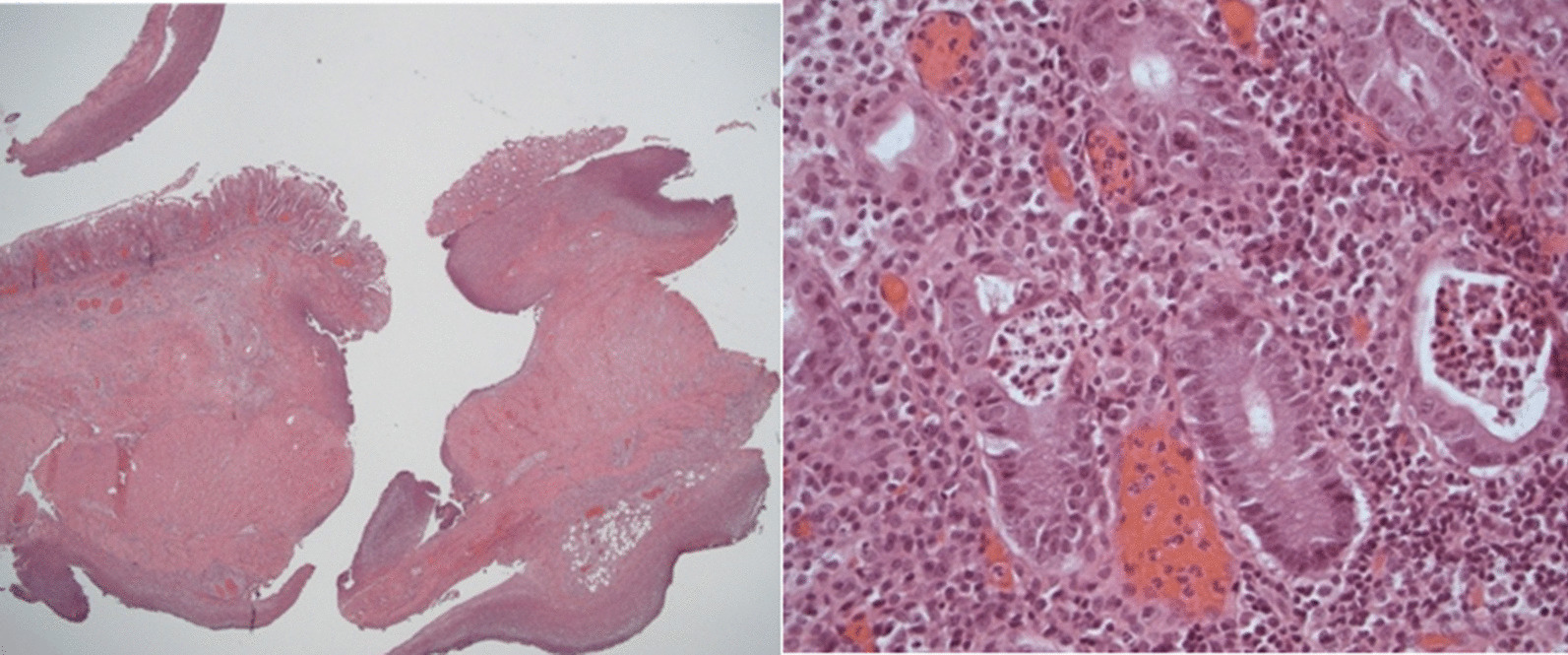
Microscopic features of the cecal perforation. Left: a transmural perforation shows fibrinopurulent substance on the peritoneal surface (HE ×25). Right: the mucosa displays moderate number of lymphocytes and plasma cells infiltration as well as cryptitis (neutrophils infiltrating in glandular cells) (HE ×400)

The patient underwent emergent exploratory laparotomy, which revealed sigmoid colon perforation of about 0.5 cm. Enterolysis and repair of sigmoid colon were performed. Pathology demonstrated active enterocolitis with ulceration, cryptal microabscess, transmural colonic perforation, and peritoneal reaction (Fig. 3). There was no evidence of chronic mucosal injuries such as granulomas or low-grade nuclear dysplasia, arguing against an underlying diagnosis of inflammatory bowel disease. Intraoperative stool specimen nucleic acid amplification testing had turned positive for *Campylobacter* spp., with negative results for other bacterial pathogens, whereas a stool obtained at admission and blood cultures remained negative. He was treated with 8 days of vancomycin, metronidazole, and ceftriaxone. However, ascites culture collected during operation reported growth of *Escherichia coli*, which was resistant to ceftriaxone, this corresponds to the high prevalence of fecal carriage of nonsusceptible *E. coli* in children in Taiwan. [2] Therefore, we substituted meropenem for treatment.

Postoperatively, he was admitted to the pediatric intensive care unit for close monitoring and given total parenteral nutrition. Improving leukocytosis and decreasing CRP occurred in the following days. His symptoms improved and he tolerated the diet well. He was discharged 15 days after hospital admission. He was evaluated in the clinic a week after discharge with significant improvement in his symptoms and surgical wound.

## Discussion

*Campylobacter* spp. is a leading cause of infectious gastroenteritis that is usually self-limited. Although *Campylobacter* spp. infection can result in a variety of complications, rarely does it cause intestinal perforation. To date, there are only a handful of cases of spontaneous intestinal perforation caused by *Campylobacter* infection. [3–11] Here (Table 1) we summarize each case, including basic information and notable features. To summarize, the most common perforation site is the cecum (5 out of 10 cases); the perforations mostly preceded with sudden worsening abdominal pain, concurrent fever, and leukocytosis; and colonic dilatation (8 out of 10 cases) and toxic megacolon (7 out of 10 cases) are the ominous signs of intestinal perforation in the majority of the cases, although the latter did not present in our case.

**Table 1:** Summary of reported cases of spontaneous intestinal perforation caused by *Campylobacter* infection

	Basic profile	Perforation site	Special feature
Vyas *et al.* (1993)	38 year old male	Cecum + Sigmoid	Toxic megacolon
Larvol *et al.* (1994)	38 year old female	Transverse colon	Toxic megacolon
Kummer *et al.* (1998)	53 year old male	Colon	Toxic megacolon
Jackson *et al.* (1999)	50 year old female	–	Toxic megacolon
Cooke *et al.* (1999)	24 year old female	Cecum	
Fang *et al.* (2000)	5 year old male	Cecum	Appendicitis + colon dilation
	3 year old male	Sigmoid colon	Toxic megacolon
Jassim *et al.* (2011)	80 year old female	Terminal ileum	Elevated CRP (33 mg/L)
Fischer *et al.* (2013)	20 year old male	Cecum	Elevated CRP (264 mg/L) No toxic megacolon
Jain *et al.* (2019)	32 year old male	Cecum	Toxic megacolon

There are several unusual features in our case. Firstly, the site of the perforation is not typical. Secondly, the perforation was not preceded by a toxic megacolon. To our knowledge, this is the first campylobacter intestinal perforation case located in the sigmoid colon and without toxic megacolon. However, our case had markedly elevated CRP (39.57 mg/dL), which is compatible with the cases of Jassim *et al.* [7] and Fischer *et al.* [6].

When it comes to intestinal perforation, there are numerous etiologies and risk factors that can result in full-thickness injury of the bowel wall, ranging from instrumentation (for example, endoscopy, instillation of contrast, cautery application during surgery), trauma (blunt or penetrating), bowel obstruction, and neoplasms (particularly colon carcinoma). However, in the case of sigmoid colon perforation, the cause of perforation is mainly from iatrogenic tools [12], foreign body [13], trauma [14], diverticular disease [15], and sometimes stercoral [16]. There is only a rare case of sigmoid colon perforation resulting from infectious colitis and it is even rarer when it was caused by *Campylobacter* spp.

Nevertheless, *Campylobacter* spp. was not the only cause of the perforation. Septic shock, which our patient experienced at the beginning of his course, can reduce the blood flow to the intestines (occlusive or nonocclusive mesenteric ischemia) for an extended period of time and increases the risk of perforation. Also, the perforated site was close to the Sudeck’s critical point, which is described as the point of origin of the last sigmoidal artery from the inferior mesenteric artery [17]. This point is relatively avascular and considered as a watershed line. Spontaneous perforation in our patient may be secondary to ischemia at this Sudeck’s critical point due to the combination of septic shock and reduced venous return caused by colonic dilatation. Although the ascites culture grew ceftriaxone resistant *E. coli*, we do not deem it as the major factor of the intestinal perforation owing to its fecal carriage rate in children. Last but not least, antimotility agents taken by our patient prior to admission may worsen the infection, and there is evidence that they can prolong the duration of fever, diarrhea, and excretion of the organism in some types of dysenteric illnesses [18]. This serious condition can also be worsened by the use of steroids and chronic medical problems [19–21].

## Conclusions

We presented this case because of the rarity of *Campylobacter*-induced sigmoid colon perforation in the pediatric population. We consider the risk factors such as septic shock-induced ischemia, *Campylobacter* spp. infection, and usage of antimotility agent to be associated with the perforation of the sigmoid colon. It is important to keep in mind that there is a possibility of colonic perforation in cases of infectious colitis caused by *Campylobacter* with similar conditions to our case. Infectious colitis caused by *Campylobacter* should be managed cautiously, and the use of antimotility agents in such conditions should be considered judiciously.

## Data Availability

All data generated or analyzed during this study are included in this published article.

## Abbreviations

CRP: C-reactive protein
*E. coli*: *Escherichia coli*
